# Multiple myeloma acquires resistance to EGFR inhibitor via induction of pentose phosphate pathway

**DOI:** 10.1038/srep09925

**Published:** 2015-04-20

**Authors:** Yan Chen, Ruibin Huang, Jianghua Ding, Dexiang Ji, Bing Song, Liya Yuan, Hong Chang, Guoan Chen

**Affiliations:** 1 Department of Haematology, The First Affiliated Hospital of Nanchang University, Nanchang 330006, China; 2 Department of Haematology, Jiangxi Academy of Medical Science, Nanchang 330006, China

## Abstract

Multiple myeloma (MM) was characterized by frequent mutations in KRAS/NRAS/BRAF within the EGFR pathway that could induce resistance to EGFR inhibitors. We here report that EGFR inhibition solely exhibited moderate inhibition in KRAS/NRAS/BRAF wildtype (triple-WT) MM cells, whilst had no effect in myeloma cells with any of the mutated genes. The moderate inhibitory effect was conferred by induction of pentose phosphate pathway (PPP) when cells were treated with Gefitinib, the EGFR inhibitor. Combination of Gefitinib with PPP inhibitor 6AN effected synergistically in triple-WT cells. The inhibition could be restored by addition of NADPH. Dual EGFR/ERBB2 inhibitor Afatinib also exhibited similar effects. Further genetic silencing of EGFR, ERBB2 and mTOR indicated that major effect conferred by ERBB2 was via convergence to EGFR pathway in MM. Our results contributed to the individualized targeted therapy with EGFR inhibitors in MM.

Identification of driver mutations in multiple myeloma (MM) holds great promise for personalized medicine, whereby patients with particular mutations would benefit from appropriate targeted therapy^1^. Two recent studies have addressed the genomic landscape of MM and have provided unprecedented insight into MM^2,3^. The studies identified frequent mutations in KRAS (particularly in previously treated patients), NRAS, and BRAF. Mutations were often present in subclonal populations, and multiple mutations within the same pathway (e.g., KRAS, NRAS, and BRAF) were observed in the same patient^3^.

These observations therefore gather attention to some of the current trials investigating the role of EGFR inhibitor in MM, as activating mutations in KRAS/NRAS/BRAF is believed to confer resistance to EGFR inhibition^4^,^5^,^6^. In colorectal carcinoma, numerous clinical studies have shown that anti-EGFR therapies are effective only in a subset of patients with colorectal cancer. Mutations in the KRAS and BRAF genes have been confirmed as negative predictors of the response to EGFR-targeted therapies^7^,^8^,^9^. Nonetheless, the role of KRAS/NRAS/BRAF mutations in MM in relation to anti-EGFR therapy has yet to been elucidated.

Interestingly, myeloma patients present a variety of clinical courses and survival. As an incurable disease, the underlying genetic and genomic diversity classifies patients with notably better or worse prognosis^10^,^11^,^12^,^13^. Whether those phenotypes are associated with certain genotype remains an interesting subject. Unlike most tumour types exhibiting mutation in genes within RAS gene family, in which solely one gene (e.g. KRAS) is mutated predominantly^14^,^15^,^16^, MM showed relatively equal frequencies of KRAS and NRAS mutations^17^,^18^. Therefore, MM features a unique model to study the mutations within RAS family and sensitivity to anti-EGFR inhibitors.

In the current study, we aimed to provide insight to the individualized anti-EGFR regime in MM by in silico analysis the Genomics of Drug Sensitivity in Cancer (GDSC), and test our hypothesis that solely KRAS/NRAS/BRAF triple-wildtype (WT) subjects could primarily benefit from anti-EGFR treatment. Also, we studied the metabolic shift in this triple-WT subtype to exploit the therapeutic role of combination of anti-metabolism with EGFR inhibition.

## Results

### Mutations in EGFR pathway components are associated with drug resistance

**Figure 1:**
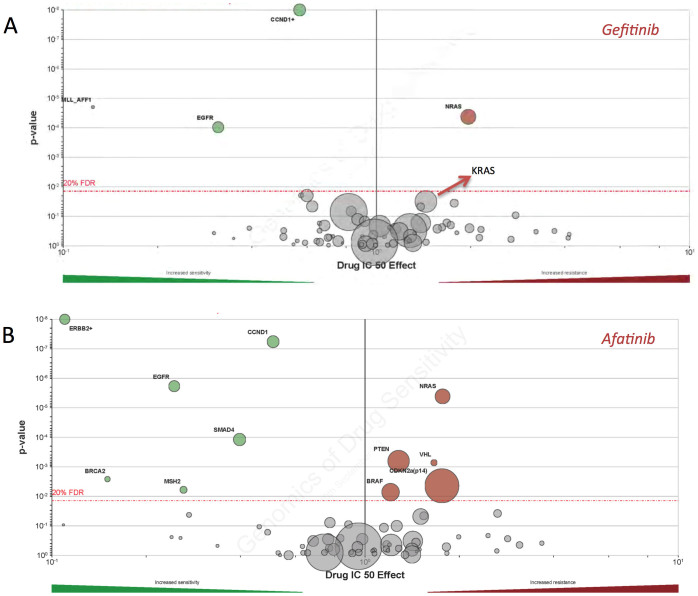
Mutations in KRAS/NRAS/BRAF conferred resistance to EFGR inhibitors. Reproduction of the Genomics of Drug Sensitivity in Cancer (GDSC) database generating the volcano plots. Green and red circles respectively encompassing sensitive and resistant cells with certain mutated gene passing 20% false discovery rate (FDR) with size of the circles indicating cell line numbers, showing mutations in KRAS/NRAS/BRAF conferred resistance to A) Gefitinib and dual EGFR/ERBB2 inhibitor and B) Afatinib in a variety of cancer cells.

It has been reported that mutations in KRAS was associated with resistance to EGFR inhibitors. As the EGFR inhibitors is currently in clinical trial for potential benefit in MM patients, we aimed to address the role of mutations in common components of EGFR pathway in MM. By further mining of the data by Lohr et al^2^, we noticed that EGFR mutation per se occurred solely in 2% of patients of whom many also harboured NRAS mutations. Of note mutations in KRAS, NRAS, and BRAF occurred in mutual exclusivity, indicating the compensatory role of each mutant gene. In all, there were up to 45% of patient with at least one mutated genes, indicating that such population could be primarily resistant to EFGR inhibitors. We then looked at the individual mutations in the cohort and found that all mutations were located in the exon and most mutations were documented in previous reports as activating mutations, which further supported our speculation (data not shown). We then exploited the GDSC database and found that in a variety of cancer cells, mutations in KRAS, NRAS, and BRAF were associated with resistance to common EGFR inhibitors like Gefitinib and Afatinib, in spite of some mutations that did not pass false discovery rate (FDR), possibly due to complexity of genetic background throughout so many cancer types (Fig. 1A–B; Suppl. Fig. 1A–B).

### Metabolic shift confers resistance to KRAS/NRAS/BRAF WT myeloma cells

**Figure 2:**
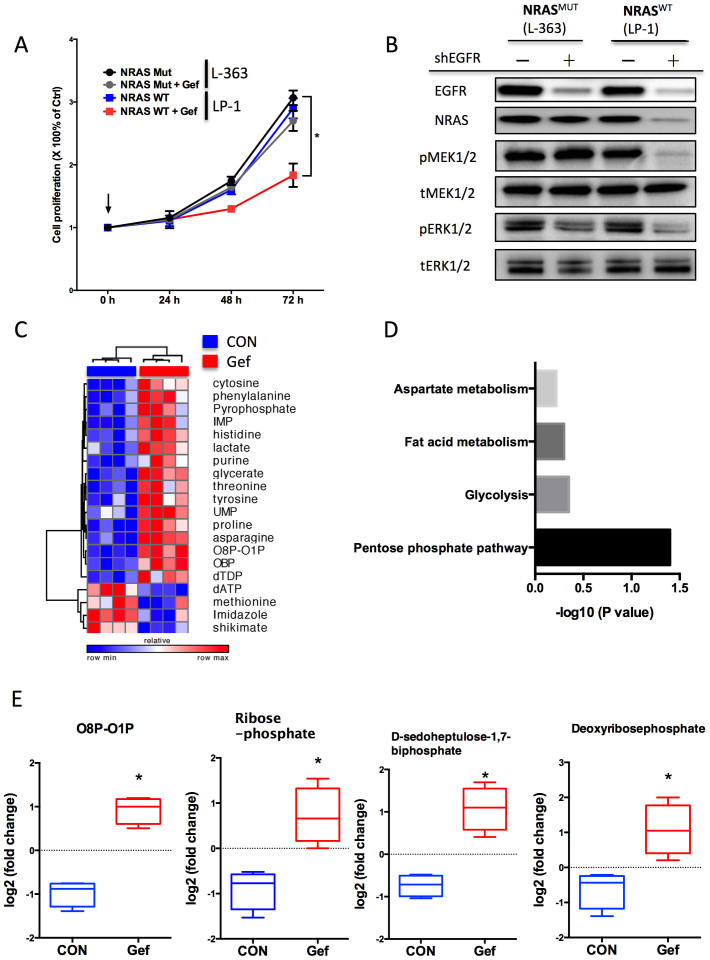
EGFR inhibitor was effective for triple WT MM cells. A) Gefinitib (5 μM) exhibited moderate inhibition in NRAS WT myeloma cells but not in mutated cells; B) Mutated NRAS was able to activate downstream effectors without EGFR signalling; C) metabolic shift of NRAS WT myeloma cells (LP-1) treated or untreated with Gefitinib (5 μM) for 24 h. Heatmap showing top changed metabolites between groups (each column representing a replicate within group, n = 4). D) MSEA showing significant change in metabolites within pentose phosphate pathway with E) representative metabolite levels in LP-1 cells.

**Figure 3:**
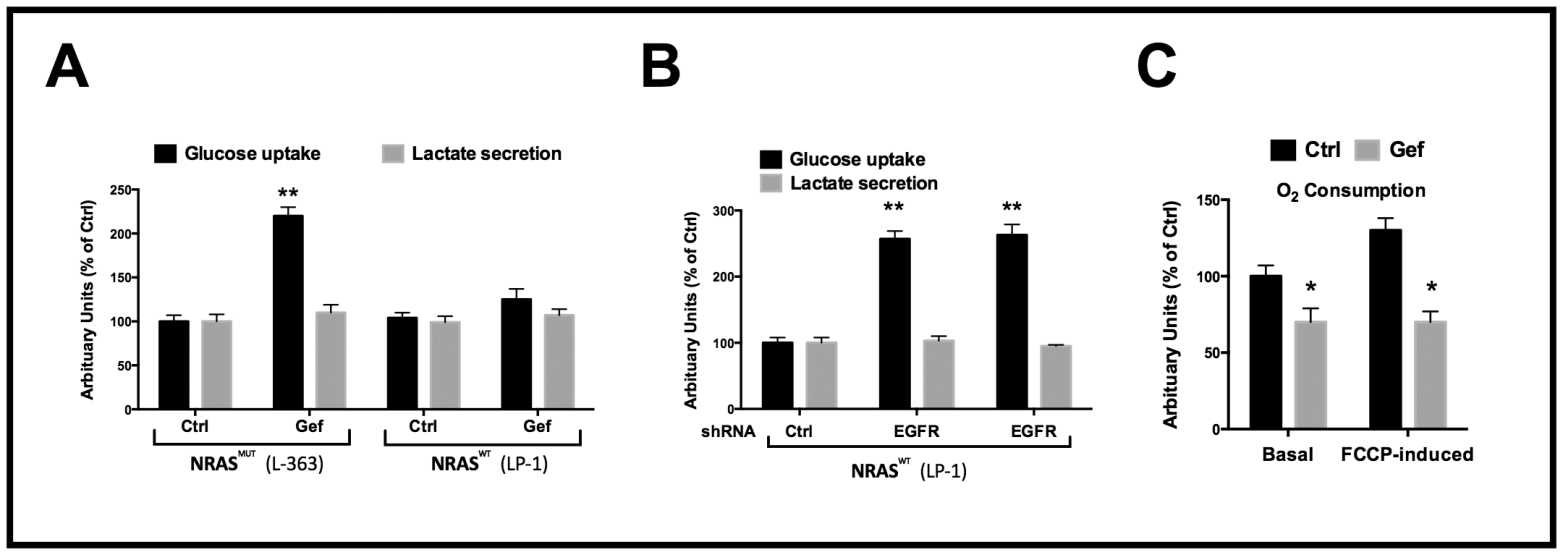
EGFR inhibition enhances glucose uptake and suppresses oxygen consumption. A) glucose uptake and lactate secretion measured by YSI in NRAS Mut and WT myeloma cells treated with Gefitinib (Gef, 5 μM) or control for 24 h or B) infected with shRNAs against the (Middle: shEGFR#1; Right: shEGFR#2). Bars, average of 3 independent samples ± SD, **P < 0.01; C) intact cellular respiration measured using the Seahorse Bioscience XF24 analyzer, under basal conditions or in the presence of FCCP in triple WT myeloma cells treated with Gefitinib (5 μM) for 24 h. Levels of oxygen consumption were normalized to cell number. Bars, average of 3 independent experiments ± SD. *P < 0.05.

Though EGFR inhibitors have shown promise in the clinical practice against some cancers, adaptive resistance remains a major problem. We therefore tended to study the metabolic shift in myeloma cells with KRAS/NRAS/BRAF WT background in response to EGFR inhibition, which was expected to confer primary efficacy. As expected, NRAS Mut cells were primarily resistant to EGFR inhibition, compared with NRAS WT cells (Fig. 2A). The NRAS Mut cells were able to activate downstream elements without EGFR signalling (Fig. 2B). Similar results were also obtained in cells with different KRAS and BRAF status (Suppl. Fig. 2A–B). Nonetheless, inhibitory effect upon KRAS/NRAS/BRAF WT cells was not lasting, and cells were not dying in the presence of EGFR inhibition (data not shown). We thus performed metabolic profiling in KRAS/NRAS/BRAF WT (triple WT) cells treated or untreated with Gefitinib and found significant increased metabolites from the pentose phosphate pathway (PPP) in cells with EGFR inhibition (Fig. 2C–E). Such metabolic shift was not seen in KRAS/NRAS/BRAF mutated cells with EGFR inhibition (Suppl. Fig. 2C). In the confirmation assays, we noticed increased glucose uptake and unchanged lactate secretion in NRAS WT cells and both substances unchanged in NRAS Mut cells (Fig. 3A). Similar results were also recapitulated in cells with genetic silencing using 2 shRNAs against EGFR (Fig. 3B). In accordance, cellular oxygen consumption was not notably changed in WT cells treated with Gefitinib (Fig. 3C). Here we provided evidence that EGFR inhibition, even in theoretically selected cells, could not provide lasting effects due to adaptive metabolic shift to PPP.

### Combination of PPP and EGFR inhibition synergistically suppressed triple WT myeloma cells

**Figure 4:**
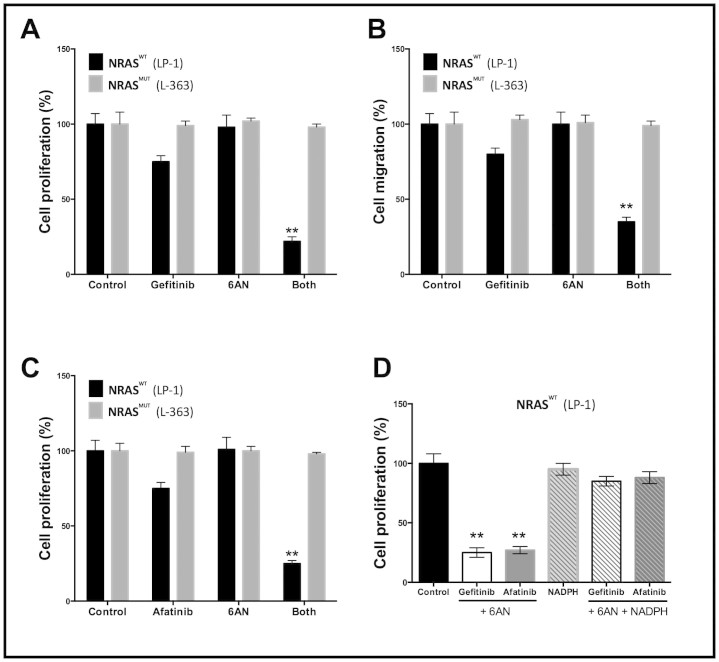
Combination of EGFR and PPP inhibitors exert synergistic effect in triple WT MM cells. Both A) cell proliferation and B) migration were significantly supressed with drug combination (both treated at 5 μM of dose for 48 h); C) Combination with EGFR/ERBB2 dual inhibitor Afatinib (5 μM) also showed synergistic effects; D) The inhibition by combination treatment was recovered almost completely by supplement of NADPH.

**Figure 5:**
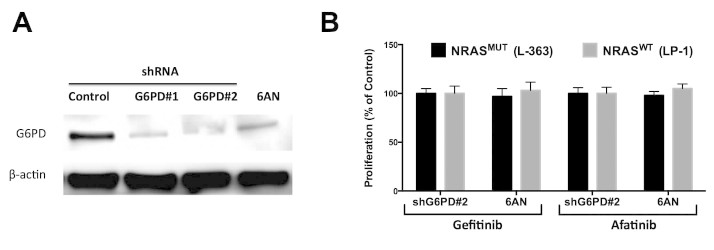
Genetic inhibition of G6PD showed similar effects to 6AN. A) LP-1 cells transfected with G6PD shRNA showed decreased G6PD to a similar extend to 6AN; B) Combination of Gefitinib or Afatinib with either genetic G6PD silencing or 6AN showed similar effects on cell proliferation.

**Figure 6:**
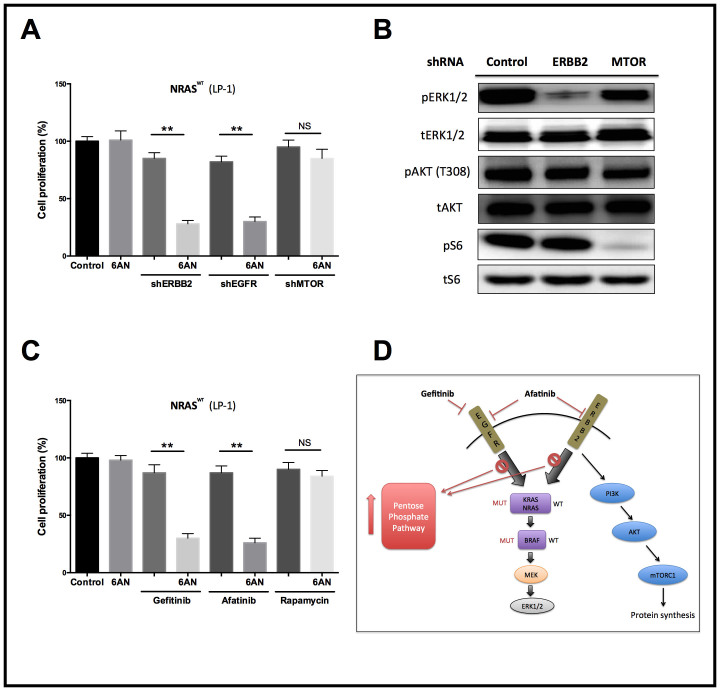
Afatinib conferred synergistic inhibition with 6AN via inhibition of EGFR pathway. A) Genetic silencing of ERBB2, EGFR, and mTOR respectively in NRAS WT myeloma cells showing no effect of mTOR inhibition in combination treatment; B) Blockade of ERBB2 in myeloma cells did not change downstream effector of mTOR but solely downstream of EGFR; C) Pharmaceutical inhibition of mTOR using rapamycin did not show synergy with 6AN as compared with Gefitinib and Afatinib; D) Scheme of how triple WT MM cells respond to EGFR inhibition and metabolic shift in response.

With aforementioned results, we speculate addition of PPP inhibitor in the setting of triple WT cells. Combination of 6AN not only synergistically inhibited the proliferation of triple WT myeloma cells but also decreased the migratory capacity (Fig. 4A–B). Similar effects were also acquired using the EGFR/ERBB2 dual inhibitor Afatinib (Fig. 4C). As it has been reported that 6AN effects with the production of intracellular reactive oxygen species (ROS), which impeded NADPH generation, we supplemented the triple WT cells with NADPH and the inhibition was restored, whereas the effect was not observed in cells with KRAS/BRAF/NRAS mutations (Fig. 4D; Suppl. Fig. 3A–B). To further validate our findings, we found that genetic silencing of G6PD, a key enzyme in PPP reached similar inhibitory effects to 6AN in combination with EGFR inhibitors (Fig. 5A–B). Both EGFR and PI3K/mTOR pathways were critical downstream signalling routes for ERBB2, a target that Afatinib inhibited. We then tended to elucidate via which pathway Afatinib was effecting in the setting of MM. Using genetic silencing of mTOR, ERBB2, and EGFR, we observed that mTOR activity was basically unchanged when ERBB2 was silenced, and the synergistic effect was not recapitulated with the presence of rapamycin (Fig. 6A–C). In summary, we have shown that combination of EGFR and PPP inhibition could synergistically inhibit triple WT MM cells via NADPH depletion. The dual ERBB2/EGFR inhibitor also effected majorly via EGFR pathway in myeloma cells.

## Discussion

EGFR inhibitors, such as Gefitinib and Afatinib have been wildly used clinically in certain types of cancer that have activation of EGFR pathway, although selective sensitivity and lasting effect in sensitive cases remain critical problems^19^,^20^. We report here that anti-EGFR treatment have no effect in KRAS/NRAS/BRAF mutant MM cells, and have moderate inhibition in triple WT cells, as reported in colorectal cancer. We hypothesized that metabolic compensatory mechanisms are activated when EGFR signalling is suppressed by either pharmaceutical or genetic silencing in triple WT cells, thereby preventing a more complete therapeutic response. Metabolite profiling revealed striking changes in the metabolome of triple WT MM cells treated with Gefitinib, including increased levels of PPP intermediate metabolites. The pentose phosphate shunt, which is often upregulated in cancer, has both biosynthetic and oxidative functions, representing the main source of NADPH via its oxidative branch and supplying ribose-5 phosphate for nucleotide synthesis. With restoration by supplement of NADPH, we speculate that triple WT myeloma cells may be particularly vulnerable to loss of reducing power, a mechanism not yet been revealed and warrants further investigation. Therefore, these data suggest that the PPP becomes a crucial metabolic survival mechanism triple WT MM cells when they are adapting suppressed EGFR signalling. To test this hypothesis, we used the antimetabolite 6AN, which inhibits the PPP dependent NADPH supply^21^, in combination with Gefitinib. The Gefitinib/6AN combination dramatically suppressed the proliferation of triple WT cells, which was rescued by supplementation of NADPH. Similar results were seen with Afatinib, a dual inhibitor for EGFR and ERBB2^22^. Afatinib has been tested in a clinical trial against HER2 positive breast cancer cells and can be used as a TKI-PET tracer^23^, further underscoring the links between the metabolic status of tumour cells and their response to PPP inhibition. Notably, it has been reported that combination of chloroquine and 6AN triggered activation of the NF-κB and inflammasome pathways in a TSC2-dependent manner and induced intracellular ROS in the setting of hyper-active mTOR^24^. We have not noticed such change in our study (data no shown). This however corresponds with our observation that the ERBB2 signalling in MM cells mainly converges towards EGFR rather than the PI3K/mTOR pathway (Fig. 6D). Of note, PPP does not only help in stress situations by providing NADPH, but also through maintaining carbon homoeostasis and as regulator of stress-induced gene expression^25^,^26^. Transient activation of the PPP is a metabolic signal required for balancing and timing gene expression upon an oxidative burst, and changes in a panel of anti-oxidative genes supplements the scenario where NADPH is not present. Whether secondary PPP activation in MM had similar effects appears to be interesting.

Although metabolic shift towards PPP is noted in the current study, formulation of culturing media could impact on metabolism as well. RPMI-1640 with L-Glutamine, which is a common media for haematological cells. It contains 2g/L of D-glucose and 0.3g/L of L-Glutamine. Compared with normal human blood sugar level (0.8–1.2g/L) the RPMI represented a borderline diabetic phenotype^27^. It has been reported that both acute and chronic elevation of glucose level in culturing media increase PPP activity in astroglia, in which PPP works as a preventive mechanism against ROS in brain^28^. Although in our study, both treatment and control cells subject to MSEA were cultured in the same media, metabolic profiling of cells in media with different glucose concentrations warrants further studies.

Another dilemma of our study is that in vitro studies could not simulate clonal heterogeneity of MM that was found to play a role in vivo^3^. Of note, point mutations in the most significantly mutated genes are found to be clonal in some patients but subclonal in others. Namely, these mutated genes could both initiate and potentiate MM in different population. BRAF mutations are found more often to be subclonal and, in some cases, coexistent with NRAS or KRAS mutations. In our reproduction using the cBioPortal platform, we could not display the heterogeneity. The evolutionary convergence within the same pathway has been addressed in some cancers^29^,^30^. These results also have important clinical implications for MM clinical trials. For example, BRAF inhibitors are being explored in MM harbouring a BRAF mutation, and the first patient with BRAF V600E-positive MM who experienced a durable response to BRAF inhibition has just been reported^31^. However, if a BRAF mutation is not clonal, suboptimal clinical benefit would be expected. In principle, treating patients harbouring subclonal BRAF mutations with BRAF inhibitors may stimulate the growth of BRAF-wild-type tumour cells. Combined BRAF and MEK inhibition might mitigate this effect, but this remains to be demonstrated in vivo. In our study, we respectively tested cells with any of the KRAS/NRAS/BRAF mutations and have discovered a drug combination for triple WT cells. However, such therapy targeting a subtype present in only a fraction of tumour cells would be expected to affect only that subclone, leading to limited clinical benefit, plus our findings that the combination had no effect against MM cells with any mutation.

The translational potential of our study lies in the identification of a subgroup of MM patients that could potentially benefit from EGFR inhibition and prolonged effect could be expected when combined with metabolic inhibition. One of the reasons that current targeted therapies against a variety of cancers lack lasting effects is that genetic/genomic classification of tumours usually depends on the effect of mutation in a single gene. As shown in the current study, even a triple-WT subtype of MM showed only moderate response to EGFR inhibitors, underscoring complex compensatory mechanisms shunting the targeted pathway. Given the aforementioned subclonal heterogeneity in the MM context, it remains at large to pinpoint a subgroups of MM patients that would be curable to a certain targeted regime or combination. Therefore, our next step is to enumerate and simulate the extent of clonal heterogeneity in vitro, to develop more specified targeted therapy and to interpret the results of subsequent therapy in light of such genetic heterogeneity. Effective targeted therapy will require either drug combinations targeting distinct subclones or, more likely, deployment of targeted therapies only in patients for whom the drug target is entirely clonal.

## Methods

### Cell lines

LP-1 (ACC-41), L-363 (ACC-49), RPMI-8226 (ACC-402), and U-266 (ACC-9) myeloma cells were originally obtained from Leibniz-Institut DSMZ-Deutsche Sammlung von Mikroorganismen und Zellkulturen GmbH (DSMZ) and were confirmed by COSMIC database and PCR to harbour specific or no mutations in KRAS/NRAS/BRAF, respectively, as shown in Table 1. All cells were cultured in RPMI-1640 supplemented with 10% FBS, 100 mg/mL of penicillin, and 100 mg/mL of streptomycin.

### Drugs and shRNA

Gefitinib (Gef) and Afatinib were obtained from, and 6-aminonicotinamide (6AN) was obtained from Sigma-Aldrich. Short hairpin RNAs (shRNA) against EGFR (TRCN0000039635 and TRCN0000121069), ERBB2 (TRCN0000382352 and TRCN0000010341), mTOR (TRCN0000195453 and TRCN0000197150), G6PD (TRCN0000221356 and TRCN0000221353) or control (shGFP) were obtained from the RNAi Consortium (TRC).

### Nutrient and oxygen consumption

Metabolite levels in the medium were measured using the Yellow Springs Instruments (YSI) 7100 as previously described^32^. Oxygen consumption was measured under basal conditions or in the presence of the Trifl uorocarbonylcyanide phenylhydrazone (FCCP) using the Seahorse Bioscience XF24 analyzer as previously described^33^. Levels of oxygen consumption were normalized to cell number.

### MTT assay

MTT (3-[4,5-dimethylthiazole- 2-yl]-2,5-diphenyl tetrazolium bromide) was used to study the cell viability and proliferation. Briefly, cells were seeded in 96-well plates. After 24 hours, cells were treated with Gefitinib, Afatinib, or 6AN (all at 5 μM). In addition, cells with equivalent volume of dimethylsulfoxide (DMSO) was served as control. After 48 h of incubation, medium of all wells were exchanged with fresh medium and cells were kept for 24 h of incubation. Medium of all wells were then removed and 50 μl of 2 mg/ml MTT dissolved in PBS was added to each wells followed by incubation of 4 h. After removing content, 200 μl of pure DMSO was added to each well to dissolve blue formazan precipitate. Then, 25 μl Sorensen's glycine buffer was added. Finally, plates were read at 570 nm of absorbance with ELISA plate Reader and reference wavelength was 630 nm. The cell viability was expressed as a percentage of the control.

### Western blotting

Antibodies against KRAS, NRAS, BRAF, phosphor- and total MEK, phosphor- and total ERK1/2, phosphor- (T308) and total AKT, phosphor- and total S6, and GAPDH were obtained from Abcam. For immunoblot analyses, cells were washed with PBS and harvested in NP-40 lysis buffer (Invitrogen, Camarillo, CA). Whole-cell lysates were resolved by electrophoresis, and proteins were transferred onto polyvinylidene difl uoride membrane, blocked in Tris-buffered saline Tween-20 buffer, and probed with the indicated antibodies in this buffer.

### Metabolic profiling

Metabolite collection was performed according to the established protocol. Briefly, all extractions were done using ice-cold reagents, pre-chilled tubes and pre-chilled centrifuges at 2,000 g for 15 min. A Refrigerated speed vac was used to evaporate the supernatant and precipitates were resuspended with high-performance liquid chromatography (HPLC) grade water for mass spectrometry. Using the 5500 QTRAP coupled to the Prominence UFLC HPLC system, the selected reaction monitoring (SRM) covered a total of a total of 250 endogenous water-soluble metabolites for steady-state analyses of samples. By means of electrospray (ESI) of + 4,900 V in positive and -4,500 V in negative ion modes and determination of SRM transition and total cycle time, approximately ~ 12 data points were acquired per detected metabolite. Metabolites were separated using the Thermo Scientific Accela liquid chromatography system. A ZIC-pHILIC column (Merck) was used for LC separation using gradient elution. Metabolites were then detected using a Thermo Scientific Exactive high-resolution mass spectrometer with electrospray (ESI) ionization, examining metabolites in both positive and negative ion modes. The MultiQuant v2.0 software (AB/SCIEX) was used to analyze the iron current of each metabolite and the Metaboanalyst (http://www.metaboanalyst.ca/MetaboAnalyst/) was used to provide normalized values. Heat map and hierarchical clustering were generated using the GENE-E software by Pearson correlations and the Ward method. The Kyoto Encyclopedia of Genes and Genome (KEGG) pathway database was exploited to perform the Metabolite Set Enrichment Analysis (MSEA). Forty metabolites were significantly increased in myeloma cells treated with Gefitinib and were selected for MSEA with reference to 230 metabolites. We selected metabolic sets with at least 5 compounds for metabolic pathway clustering.

### Statistical analysis

All assays were run in triplicates in 3 independent experiments. Two-tailed Student's t-test was used to compare values between 2 groups. The P value of < 0.05 was accepted as statistically significant.

## Author Contributions

In the current study, Y.C., G.C. designed the study. Y.C., R.B., Y.Y., H.C., G.C. prepared figures. D.J., B.S., Y.Y.,H.C., G.C. did statistics. Y.C., G.C. sketched manuscript. Y.C., R.B., J.D., D.J., B.S., Y.Y.,H.C., G.C. reviewed the article, and all authors approved the final version of the manuscript.

## Supplementary Material

Supplementary Information Supplementary Information

## Figures and Tables

**Table 1:** Genetic background of the cells used in the current study

Cell line	COSMIC	Tissue	Tissue sub-type	Mutation		
				KRAS	NRAS	BRAF
L-363	924239	blood	Myeloma		*	
LP-1	907791	blood	Myeloma			
RPMI-8226	905964	blood	Myeloma	*		
U-266	753615	blood	Myeloma			*
