# Author Correction: Multiple myeloma acquires resistance to EGFR inhibitor via induction of pentose phosphate pathway

**DOI:** 10.1038/s41598-023-47497-8

**Published:** 2023-11-30

**Authors:** Yan Chen, Ruibin Huang, Jianghua Ding, Dexiang Ji, Bing Song, Liya Yuan, Hong Chang, Guoan Chen

**Affiliations:** 1 Department of Haematology, The First Affiliated Hospital of Nanchang University, Nanchang, 330006 China; 2 Department of Haematology, Jiangxi Academy of Medical Science, Nanchang, 330006 China

Correction to: *Scientific Reports* https://doi.org/10.1038/srep09925, published online 20 April 2015

The Article contains an error in Figure 2, where incorrect images were used during the image assembly.

The correct Figure 2 and accompanying legend appear below.

**Figure 2:**
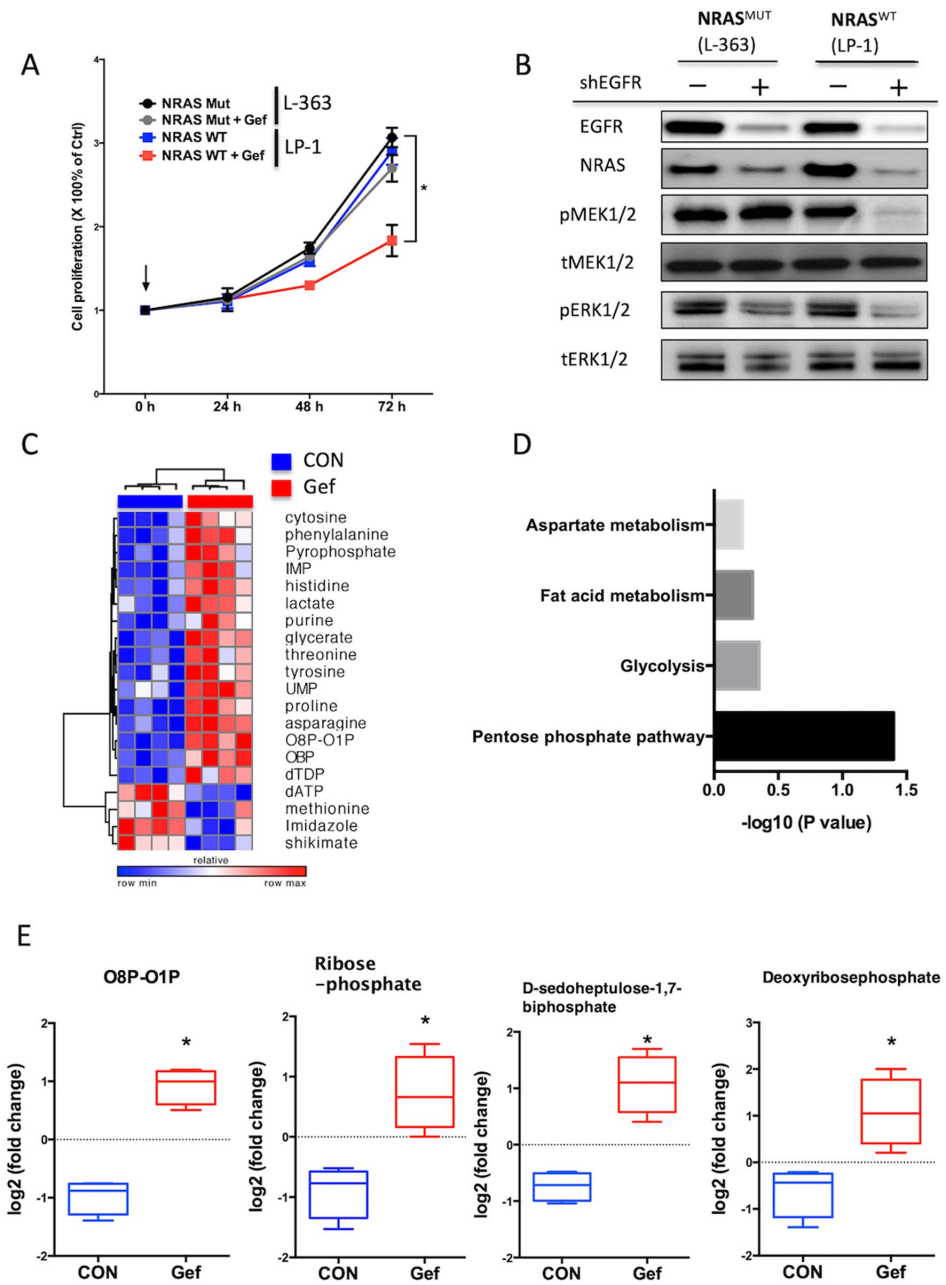
EGFR inhibitor was effective for triple WT MM cells. (**A**) Gefinitib (5 μM) exhibited moderate inhibition in NRAS WT myeloma cells but not in mutated cells; (**B**) Mutated NRAS was able to activate downstream effectors without EGFR signalling; (**C**) metabolic shift of NRAS WT myeloma cells (LP-1) treated or untreated with Gefitinib (5 μM) for 24 h. Heatmap showing top changed metabolites between groups (each column representing a replicate within group, n = 4). (**D**) MSEA showing significant change in metabolites within pentose phosphate pathway with (**E**) representative metabolite levels in LP-1 cells.

