# Supplementary material for: Multiple myeloma acquires resistance to EGFR inhibitor via induction of pentose phosphate pathway
Source: Sci Rep. 2015 Apr 20;5:9925. doi: 10.1038/srep09925 (PMC4403500; doi:10.1038/srep09925)
Supplement: Supplementary Information [file srep09925-s1.pdf]

Multiple myeloma acquires resistance to EGFR inhibitor via induction of pentose phosphate pathway

Running title: Inhibition of PPP and EGFR in MM

Yan Chen[1#], Ruibin Huang[1#], Jianghua Ding[1], Dexiang Ji[1], Bing Song[1], Liya Yuan[2], Hong Chang[1], and Guoan Chen[1*]

[1]Department of Haematology, The First Affiliated Hospital of Nanchang University, Nanchang 330006, China.

[2]Department of Haematology, Jiangxi Academy of Medical Science, Nanchang 330006, China

*Correspondence to: Dr Guoan Chen, Department of Haematology, The First Affiliated Hospital of Nanchang University, Nanchang 330006, China.

[#]YC and RW contributed equally.

Supplementary Figure 1

A

B

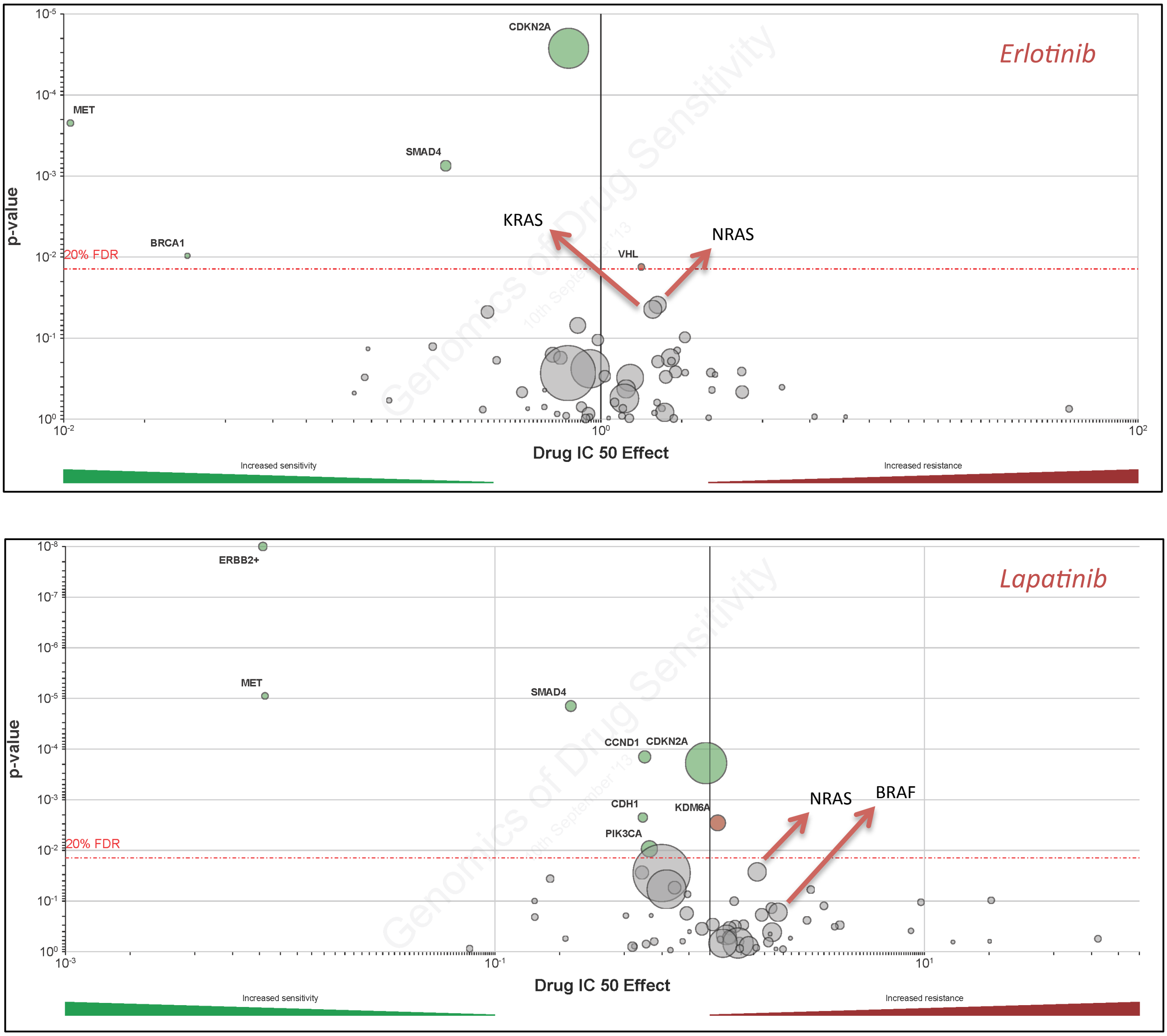

Reproduction of the GDSC database showing mutations in genes within EGFR pathway confers resistance to both EFGR inhibitors, A) Erlotinib and B) Lapatinib in a variety of cancer cells.

Supplementary Figure 2

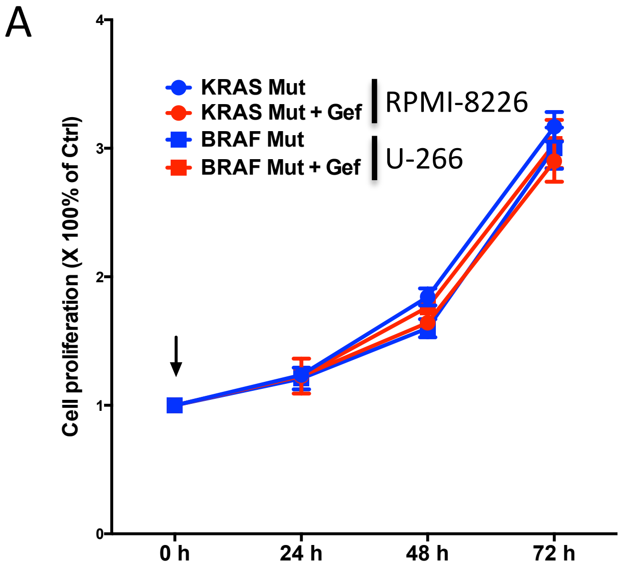

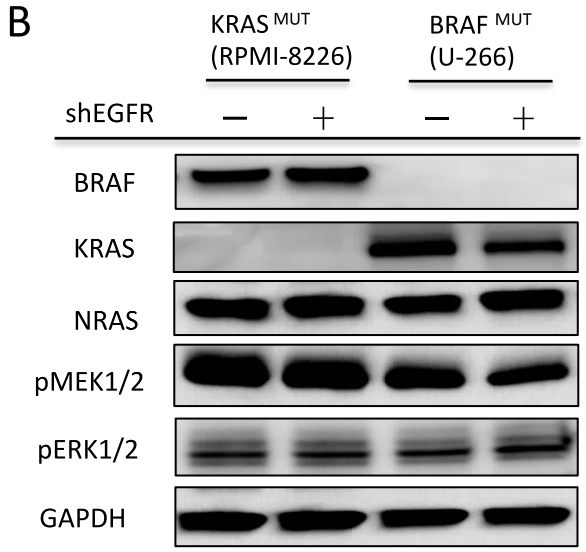

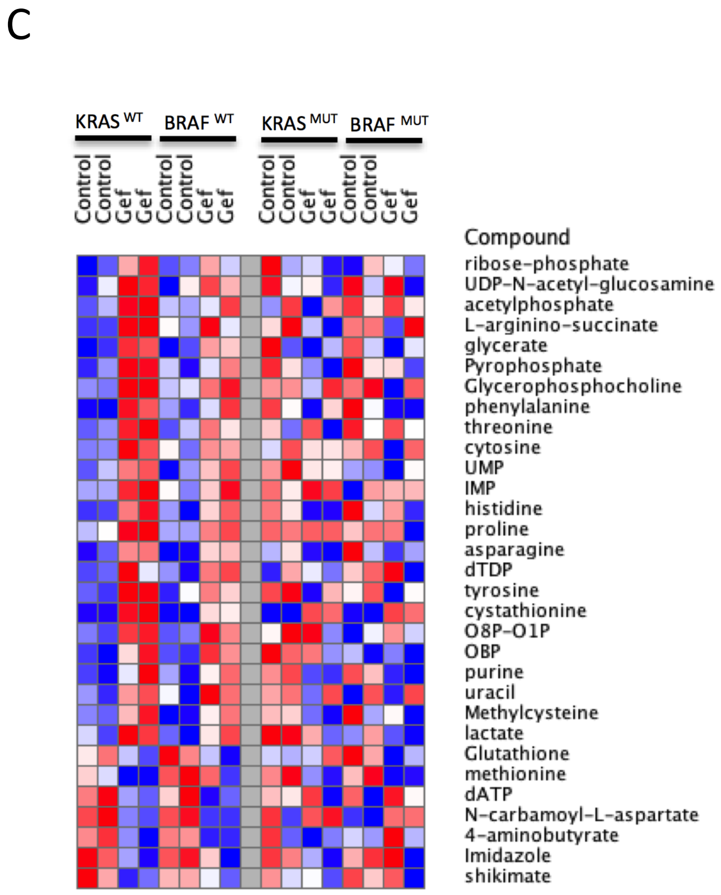

A) Mutations in KRAS and BRAF confer resistance to EGFR inhibitor Gefitinib in myeloma cells. B) Both mutations in BRAF and KRAS in myeloma are activating mutations bypassing EGFR signaling to mediate down-stream effectors. C) Metabolic profiling showing consistent changes in pentose phosphate pathway metabolites only in KRAS and BRAF wild-type cells.

Supplementary Figure 3

A

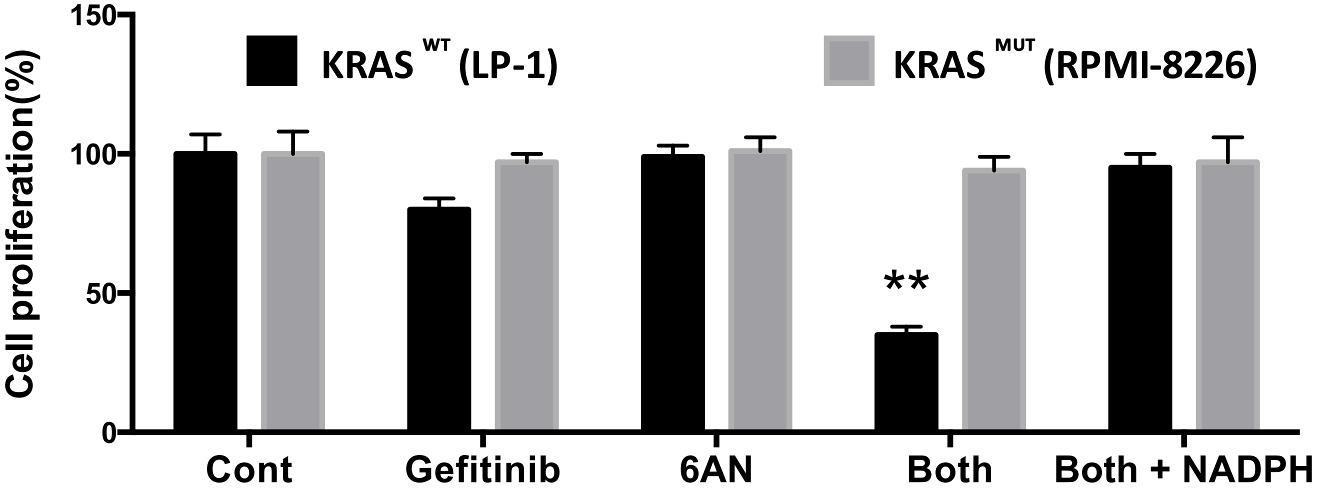

B

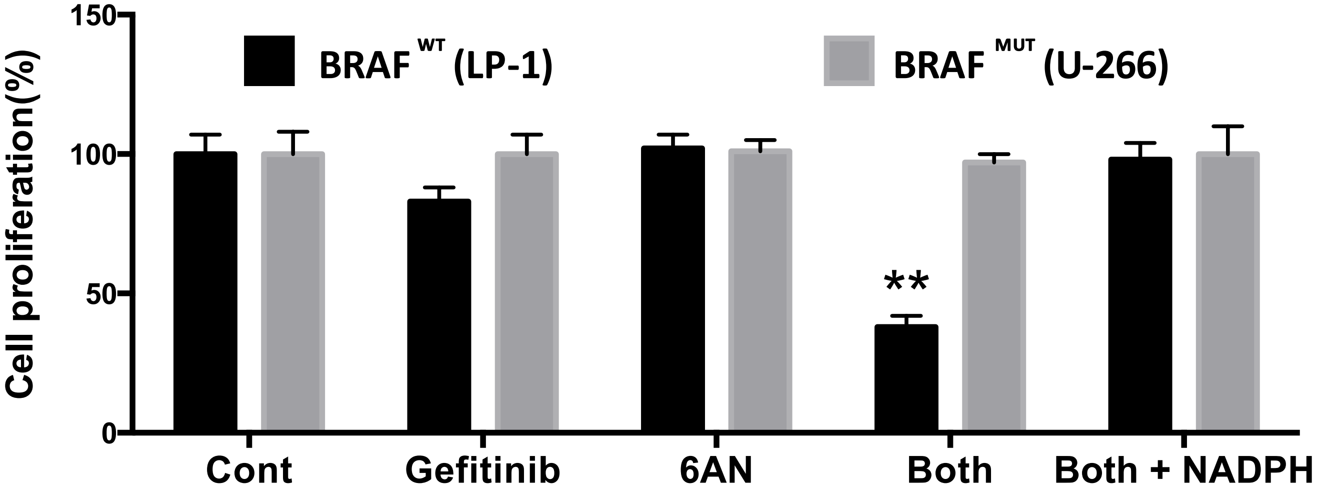

Both A) KRAS and B) BRAF mutated myeloma cells were synergistically inhibited by combination of EGFR and pentose phosphate pathway inhibitors, and were recovered with NADPH.
